# ML-DSTnet: A Novel Hybrid Model for Breast Cancer Diagnosis Improvement Based on Image Processing Using Machine Learning and Dempster–Shafer Theory

**DOI:** 10.1155/2023/7510419

**Published:** 2023-11-02

**Authors:** Mohsen Eftekharian, Ali Nodehi, Rasul Enayatifar

**Affiliations:** ^1^Department of Computer Engineering, Gorgan Branch, Islamic Azad University, Gorgan, Iran; ^2^Department of Computer Engineering, Firoozkooh Branch, Islamic Azad University, Firoozkooh, Iran

## Abstract

Medical intelligence detection systems have changed with the help of artificial intelligence and have also faced challenges. Breast cancer diagnosis and classification are part of this medical intelligence system. Early detection can lead to an increase in treatment options. On the other hand, uncertainty is a case that has always been with the decision-maker. The system's parameters cannot be accurately estimated, and the wrong decision is made. To solve this problem, we have proposed a method in this article that reduces the ignorance of the problem with the help of Dempster–Shafer theory so that we can make a better decision. This research on the MIAS dataset, based on image processing machine learning and Dempster–Shafer mathematical theory, tries to improve the diagnosis and classification of benign, malignant masses. We first determine the results of the diagnosis of mass type with MLP by using the texture feature and CNN. We combine the results of the two classifications with Dempster–Shafer theory and improve its accuracy. The obtained results show that the proposed approach has better performance than others based on evaluation criteria such as accuracy of 99.10%, sensitivity of 98.4%, and specificity of 100%.

## 1. Introduction

Unfortunately, breast cancer is one of the leading causes of death among women. In 2015, about 2.4 million people were diagnosed with breast cancer, and 523,000 of them died in 2020; the incidence has increased to 19.3 million [1]. Breast cancer is a type of cancer that begins in women's breast tissue with symptoms such as a mass in the breast, breast deformity, skin rash, discharge from the nipple, or partial scaling of the skin. To grow cancer, the gene must regulate growth and cell proliferation. These mutations will then become a mass through cell proliferation. Identifying the transporter gene of this cancer can be an essential step in predicting breast cancer. The high volume of genetic information is one of the most critical problems in representing biological molecules' large structure and function. Also, one of the most critical challenges in bioinformatics is the need to design and produce methods, algorithms, and tools to convert this large volume of often heterogeneous (low-level) data to higher-level bioknowledge [2]. Breast cancer can be effectively treated with early detection, such as a screening that detects the early initial symptoms of breast cancer using common methods such as mammography, ultrasound, and thermography, of which mammography is one of the most important early detection methods. But ultrasound or diagnostic sonography methods are more common for solid breasts because mammography is not suitable for solid breasts [3]. Because of the need for early detection, many countries have introduced screening programs. Breast cancer screening requires one or two radiologists to look at a woman's mammogram for symptoms of cancer to reduce morbidity and mortality [4]. Of course, there are errors in breast screening programs in between 15 and 35% of cancers. Because the cancer was not visible to the radiologist or he made a mistake [5].

As mentioned in [3], the most appropriate way to reduce cancer deaths is to diagnose it early so that treatment can begin. This timely diagnosis should be made reliably. Among the available methods of diagnosing breast cancer, mammography is widespread and highly accepted [6]. However, this method of diagnosing breast cancer has drawbacks. Because in some cases, there is a possibility of damage to the film or inadequate mammography image quality to diagnose the disease, which requires repeated imaging [7]. Another problem with mammographic images is that they wear out over time. Visual diagnosis of the disease from mammographic images is always erroneous and unfortunately causes between 3 and 20% error in diagnosis [8]. The masses are divided into benign and malignant. Visually, benign masses have very smooth and uniform margins. In contrast, malignant masses have dark and prominent margins, and over time, they become sharp and needle-like. Tiny calcareous particles are tiny calcium particles that appear as bright spots in mammographic images, and tiny calcareous particles are often confused with the noisy particles in the figure [9]. Due to the inherent problems of medical images, with the help of image processing, their contrast and noise are improved today. Convulsive neural networks, artificial intelligence, and machine learning are widely used in the healthcare industry and are growing rapidly [10, 11]. In recent years, there has been considerable interest in the use of artificial intelligence to complement or replace human work. In 2019, 3.8% of the articles reviewed were related to artificial intelligence [12].

## 2. Literature Review

In [13], with artificial intelligence, create a real-time breast ultrasound detection system, with quality control in the breast to improve sensitivity and specificity shortly by adding more learning data for clinical applications. The authors in [14] presented a new method for extracting prominent features of the breast based on biological data and image analysis. This information is extracted from a thermal camera. That information is used by a convolution neural network optimized by the Bayes algorithm to classify breast images as normal and suspicious. Using this proposed algorithm, 98.95% accuracy was obtained for the data of 140 people. In [15], breast cancer was diagnosed using deep learning and a combination of annular and automatic neural networks. In the experiment, the features obtained from the neural network model were used. The ridge regression method used important features of selection. Then the accuracy classification was 98.59%. In [16], by convolutional neural network and its combination with the multiscale method, the accuracy reached 97.3% [17]. Benign and malignant tumor classification from mammography images is proposed based on image processing and machine learning. In this research, region growing for segmentation and cellular neural network with a determined threshold were applied. Cellular neural network's parameters optimized for segmentation and classification with genetic algorithm. Some comparisons have been done with other methods such as Naive Bayesian, random forest algorithm, support vector machine, and K-nearest neighbor in terms of evaluation criteria such as accuracy, sensitivity, and specificity. The proposed method of this research had 96.48% accuracy, 96.87% sensitivity, and 95.94% specificity for breast cancer diagnosis. MIAS and DDSM datasets were used in this research. In [18] parenchymal enhancement is proposed for noise reduction from mammography and MRI images in breast cancer diagnosis. In [19], a review of image processing and mammography and MRI image classification has been done for breast cancer diagnosis. Microarray images for breast cancer diagnosis were proposed in [20] by using an image processing method which obtained 95.45% accuracy in detecting areas. In [20], mammography image classification for breast cancer diagnosis was proposed, which used backpropagation neural network. The accuracy of this method was estimated at 70.4% in detection and classification. Also, in [21], an overview of intelligent methods in breast cancer detection was proposed, which studied many classification methods with machine learning and image processing methods. Based on this overview, the study represented that neural network has a better rate of detecting the disease in images. Naïve Bayesian classifier based on Bayes theory in mammography images used in [22]. This paper's classification results for detection purposes are 99.11% for sensitivity, 98.25% for specificity, and 98.54% for accuracy criteria, respectively. An adaptive intelligent decision-making system was proposed in [23] for breast cancer diagnosis based on mammography images. This method is based on regression. The type of mass determines the rate of loss of life in this study, and the remaining life is predicted by mass size. In [24], a new breast cancer diagnosis method was proposed from mammography images based on feature analysis. The first part is noise reduction and image segmentation based on image processing. In the following, a classifier based on extracted features in learning is used to detect benign and malignant masses and estimate the size of masses in images. The evaluation criteria obtained 96.5% sensitivity, 89% specificity, and 95.6% accuracy.

A new method for breast cancer diagnosis based on mammography images was proposed in [25]. Low-level processing such as noise reduction, averaging, and thresholding is intended. Averaging is used for smoothing, and thresholding is used for feature extractions. Based on some features like light intensity and edges, tumor areas detected by the principles of image processing draw a rectangle around those contiguous areas separated by edges from the image and the main texture of the image, and their brightness intensities vary slightly with the use of windowing. In the following, the local mean and variance of each subwindow are separated and specified. Then the max-mean method and the least variance method identify the cancerous masses in the areas around which they are drawn out in the window section. The identification of border regions between breast tumors was performed using morphological processing and the image gradient technique. Finally, a segmentation based on morphological operators was performed that represented the tumor area. A case study based on advances in the intelligent diagnosis of breast cancer has also been studied [26]. Computer-aided design (CAD) methods have been studied based on image processing, machine learning, decision systems, fuzzy logic, and similar hybrid methods. In a different approach presented in [27], the performance evaluation of a Compton camera with Si/CZT lenses for detecting breast tumors was proposed. Using the Monte Carlo method, this simulation was performed to detect breast tumors using a Compton camera and a Si/CZT lens. Deep learning techniques [28] are used to diagnose and classify breast tumors. Three different deep learning architectures, including GoogLeNet, VGGNet, and ResNet, have been considered. An analysis has been performed between these methods. The results of this method represented that the proposed approach had high accuracy in the diagnosis and classification of tumor areas.

Visual diagnosis and evaluation of breast tumors with deep learning principles are also presented in [29]. In this way, 322 images from a clinical dataset were entered as inputs for segmentation-based clustering operations, which combine K-means and SURF algorithms. In the classification phase, a new layer was added to classify the deep learning network structure: a multiclass support vector machine. 70% of the data are considered as training, and 30% of data as a test. The improvement of the proposed approach in terms of evaluation criteria such as ROC and accuracy in detection and classification has been compared with other methods such as multilayer perceptrons neural network (MLP), decision tree, K-nearest neighbor algorithm (KNN), and support vector machine (SVM) which showed the improvement of the proposed approach over previous methods. In [30], a finite element approach based on machine learning principles for modeling the mechanical behavior of breast tissue under real-time compression conditions is presented. Also, in [31], a medical intelligent diagnosis system was presented to predict breast cancer recurrence using optimized ensemble learning. This approach, abbreviated as HBPCR, is compared to other methods such as support vector machines, multilayer perceptron neural networks, and decision trees, which show improvement of the proposed method in terms of evaluation criteria. This research's most important evaluation results were specificity with 93%, sensitivity with 77%, and accuracy with 85%. In [32], they designed a system for the initial diagnosis, examination, and treatment of breast cancer, combining the features via CNN, in which the random forest algorithm has the highest 96.65 accuracies with less error than the CNN classifier. In [33], the authors compared the architecture and accuracy of the networks and then evaluated them based on the accuracy of detection and classification and observed that CNN has a higher accuracy than MLP. In another study [34], three radiologists set criteria for evaluating the image of the title good, poor, fair, reasonable, and excellent to classify it. Now using a parallel system, they classify features using machine learning techniques such as LDA, quadratic discriminant analysis (QDA), SVM, logistic regression, and MLP, and were able to achieve an accuracy of 70 to 77 percent. Get the best 77% AUC. In [35], mammographic images were improved by medium and Gaussian filters, and the Otsu method was used to cut the breast area. They used 7,259 mammograms from the MIAS and INbreast datasets, of which 6,346 were for training and 913 were for testing. Using transfer learning, they changed the final layers of CNN. They used VGGNet, MobileNet, GoogLeNet, ResNet, and DenseNet and proposed a deep ConvNet + SVM hybrid network with an accuracy of 97.8% and an AUC of 91.4%. In [33], they tested 14 different neural networks on several databases to see which structure performed the most accurate classification on malignant cells and concluded that CNN was slightly more accurate than the multilayer perceptron neural network (MLP). They used two classification methods. One is transfer learning and the other is CNN AlexNet implementation along with a trained SVM classification by extracted features, for which an AUC = 0.86 was obtained. In [36], random forest, support vector machine (SVM), decision tree (C4.5), K-nearest neighbor (KNN), and logistic regression, methods were applied to the Wisconsin breast cancer dataset after performance evaluation. Comparing them to find the best machine learning algorithms in terms of confusion matrix, accuracy, and precision, it was found that the support vector machine with 97.2% accuracy performs better than other classifiers. In [37], three different structures of the convolutional neural network (CNN) are used to automatically detect breast cancer by analyzing tissue zones, and all three proposed architectures are tested on 275,000 images and with the results of machine learning. The proposed third architecture, which was deeper and consisted of five layers, had an accuracy of 87% and a greater amount of machine learning with an accuracy of 78%. In [38], DDSM and CBIS-DDSM databases were used and ROI was performed on 5272 images, training, and testing were performed by the AlexNet network in the form of 70−30 with an accuracy of 71.01% and an AUC of 88%. The SVM was then applied to it, increasing the result to 87.2% and the AUC to 94%. In Table 1, we review some of the above methods. The authors in [40] has proposed a system for automatic detection of machine learning algorithms and a set of different algorithms. After reviewing machine learning algorithms and different group models, experiments were performed on two datasets, and the results were compared. The results showed that the group method was superior to other methods and achieved an accuracy of 98.83%. For this reason, the proposed system is of great importance to the medical industry and the related research community. The comparison shows that the proposed method performs better than other methods. The authors in [41] present breast cancer detection from mammography images based on optimal multilevel threshold-based segmentation with DL active capsule network (OMLTS-DLCN). This model uses an adaptive fuzzy-based median filter (AFF) to remove noise and uses a multilevel thresholding algorithm based on the optimal kapur and (OKMT-SGO) algorithms for breast cancer segmentation. CapsNet-based feature extraction and backpropagation neural network classification are used for breast cancer detection. The results of tests on the Mini-MIAS and DDSM datasets show the accuracy of 98.5 and 97.55, respectively. In [42], image processing and machine learning methods have been used to diagnose breast cancer. In this article, to improve the quality of the image, the mean filter and AlexNet are used to extract features, and the relief algorithm is used to select features. In classification, MSE, SVM, KNN, random forest classifier, and the MIAS dataset were used. In [43], it first preprocesses the data and removes the noise in the mammography images, then uses machine learning methods such as support vector machine, logistic regression, and K-nearest neighbor to data classification. They use 60% of the data for training and 40% for testing. The accuracy of their proposed method is the highest at 97.7%. In [44], the performance of several machine learning algorithms such as Naive Bayes, Adaboost, XGboost, random forest, decision tree, and K-nearest neighbors on the Wisconsin Dataset has been investigated and compared. The results were tested in terms of accuracy, sensitivity, and specificity for all the above algorithms. Experimental results show that XGboost provides the highest accuracy of 98.24%.

In this study, our goal is to reduce uncertainty and increase accuracy. Uncertainty is a reason that has always accompanied the decision-maker, and it is expressed in uncertain detail in the issues. In these cases, the system parameters cannot be accurately estimated, resulting in the wrong decision. To solve the above problem, we have presented a method in this article that, with the help of Dempster–Shafer theory, reduces the ignorance of the problem as much as possible so that we can make the right decision. The remainder of this article is organized as follows in the proposed method. A new approach for breast cancer diagnosis and classification will be proposed. Then simulation results and outputs will be described, analyzed, and compared with other methods. In the end, a conclusion will be presented where a detailed evaluation of the research is made.

## 3. Proposed Method

**Figure 1:**
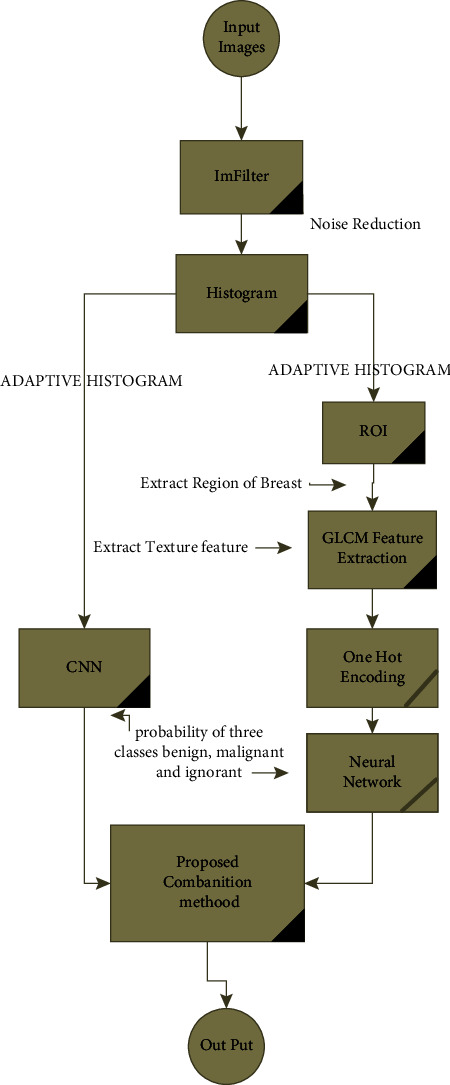
Flowchart of the proposed method.

Figure 1 shows the flowchart of the proposed method. As shown in the figure, we have used the combined method to increase the accuracy based on Shafer's theory. Classification and diagnosis of tumors for both benign and malignant classes are performed using a combination of deep learning and neural network methods. For this purpose, CNN deep neural network and MLP neural network are trained and evaluated separately for tumor diagnosis. Finally, the results of these two methods are combined using the Dempster–Shafer method. In this paper, two feature extraction methods are used. In the CNN method, the features are extracted by deep learning. In the artificial neural network, the GLCM features extracted from the images that are used. In the following steps, the probability of each class is calculated by the desired classifier. The results of the combination and the final output are created with the help of Dempster–Shafer theory. We will now describe the steps specified in the proposed method according to the flowchart.

### 3.1. Dataset

The input images used in this research are from the MIAS mini mammographic database. A British research organization obtained the data through the digitization of radiology films. These images contain 322 images of different people, for which the expert opinion of an expert has also been prepared. Images are divided into two categories: normal and abnormal, and abnormal images are classified into benign and malignant. The images are 1024 by 1024 in size and are stored in 8 bits.

### 3.2. Noise Reduction

As we know, mammographic images, due to the nature of their creation, are among the most noisy images, and to improve the final result, it is necessary to perform tweezers reduction operations on them. Accuracy in noise reduction operations can affect the results of subsequent sections such as edge detection, segmentation, and feature extraction.

Therefore, there may be points in mammographic images that are not known as salt pepper noise, Gaussian noise, or other noise, in the noise reduction stage due to their light intensity and color, which have destructive effects on the final diagnosis and classification of the type of tumor and cancerous masses. Therefore, it is necessary to perform noise reduction operations and choose a suitable and optimal method for accurately identifying these points. One of the best and most appropriate ways to reduce the noise of mammographic images, which are often peppery and salty noises or Gaussian noises, is to use a median filter [45]. This filter considers the value of the middle element of the array as the output by considering a 3 × 3 neighborhood of noise points and arranging the values of its adjacent pixels. One of the advantages of this filter is that it does not eliminate the edge of the image and does not move its position in the image (see Figure 1).

### 3.3. Histogram Equalization

Improving contrast is one of the essential things about images and will improve processing and increase accuracy. One of the best ways histogram equalization is done is on dark images, and their brightness level should be such that the important features of mammographic images, including the intended texture, can be extracted.

In the following, we describe the relation between calculating the histogram equalization [46]. For the input image (*X*), histogram *h*(*x*) is defined according to the following equation:

(1) $$\begin{array}{c} h\left(x\right)=n_{x}\;for\;x=1,2,\ldots \ldots L-1\text{,} \end{array}$$

where *n*_*x*_ is the number of observations of light intensity *x* in the image (*X*), and *L* is the last value of its light intensity. The probability of density *p*(*x*) is according to equation (2), and *N* is the number of pixels in the image.

(2) $$\begin{array}{c} p\left(x\right)=\frac{h\left(x\right)}{N}\;for\;x=1,2,\ldots \ldots L-1\text{.} \end{array}$$

Now, according to equation (2), the cumulative probability density function *c*(*x*) is calculated by the following equation:

(3) $$\begin{array}{c} c\left(x\right)=\overset{x}{\underset{k=0}{\sum }}p\left(k\right)\;for\;x=1,2,\ldots \ldots L-1\text{.} \end{array}$$

*F*(*x*) is the transfer function for histogram equalization and it maps the input image to the entire dynamic range [*x*_0_, *x*_*l*−1_] using *c*(*x*) and obtained from the following equation:

(4) $$\begin{array}{c} f\left(x\right)=x_{0}+\left(x_{l-1}+x_{0}\right).c\left(x\right)\text{.} \end{array}$$

Finally, to calculate the histogram equalization image, we use equation (5), where (*i*, *j*) is the position of the pixels in the image.

(5) $$\begin{array}{c} Y=\left\{Y\left(i,j\right)\right\}\\ =\left\{f\left(X\left(i,j\right)\right.\left|\forall X\left(i,j\right)\in X\right.\right\}\text{.} \end{array}$$

As shown in the flowchart, so far it is common to both of our proposed methods, but since we continue with two different classifications, first explain the neural network section and then the deep neural network.

### 3.4. ROI Extraction

After reducing the noise and adjusting the brightness of the output image, the desired area should be separated from the rest of the image, which contains the primary information. Then other processing should be performed on it. Additional information from radiological images such as the patient's name, unnecessary writings, and tissue should be removed, as additional information will increase processing time and may lead to errors in the final decision. In this paper, a morphological operator is used to extract the breast area following [47].

**Figure 2:**
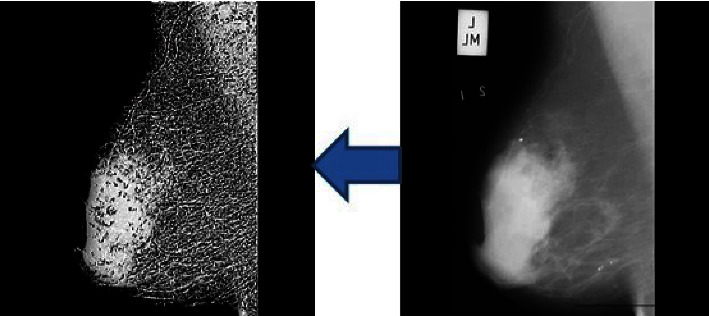
Extract the breast tissue area.

Morphology is used to change the image and expand or delete parts of the binary image by expanding and eroding. To remove the background of the image, we used the erosion operator to remove the background of the image and a flat diamond with a radius of 3.  Figure 2 shows the result of the separation of the breast tissue area with this method.

### 3.5. Feature Extraction

Most feature extraction methods are based on the spectral information of the pixels, and their helpful spatial information, such as texture, is ignored. In cases where the accuracy of our images, such as mammograms or MRI, is low and always contains noise, it is better to extract their features based on the neighborhood information of the pixels. In general, extraction methods and image texture properties are classified into four categories: statistical methods, structural, model-based extraction, and conversion-based extraction. The gray level cooccurrence matrix “Called GLCM” is one of the statistical methods for extracting texture properties by Haralick et al. in 1973 in which 23 features were presented [48] and then in 1979, the features were reduced to 8 [49]. GLCM extracts features based on the distance and angle between two pixels in a window with specific dimensions. These features include the following:

Autocorrelation, contrast, correlation, correlation, cluster, prominence, cluster shade dissimilarity, energy, entropy, homogeneity, maximum probability, sum of squares, variance sum average, sum variance, sum entropy, difference variance, difference entropy, information measure of correlation, information measure of correlation, inverse difference (INV), inverse difference normalized (INN), and inverse difference moment normalized were used.

### 3.6. One Hot Encoding

**Figure 3:**
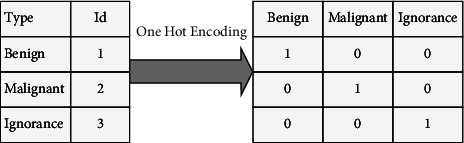
One hot encoding.

In some cases, changes to the data need to be made. These changes are usually used before the classification step to adapt the data. Therefore, it is part of the preprocessing steps. One hot encoding is used to convert nonnumeric data to numeric and can receive up to 15 items. Given that we have three classes: benign, malignant, and ignorant, we want to convert these string values into numeric values with this coding method. To do this, we create rows with the desired number of data and fill them with 0 and 1. Set the desired value in that row to 1 and the other cells in that row to zero.  Figure 3 shows an overview of the one hot method used in our paper by considering the three classes benign, malignant, and ignorant, respectively. Benign and malignant data are known according to the dataset. However, for the ignorant state, we ignore any data other than these two classes. Due to the selected dataset, normal data are considered ignorant.

### 3.7. Neural Network

An artificial neural network consists of three layers: input, hidden, and output. Each layer is composed of a group of nerve cells called neurons. The input and output layers are entirely connected to the middle layer [50]. In this section, we use the classification of a multilayer perceptron neural network or MLP with the backpropagation learning method, which is one of the most common and popular neural network structures and can produce the best outputs by choosing the correct internal structure. Its use has been observed in most medical applications such as epidemiology, predicting prostate cancer, predicting unwanted pregnancy, and predicting death after open-heart surgery [51]. The extracted feature from the image is given to the input layer of the neural network, and we use the sigmoid function to calculate the output of the hidden layer neurons and the output layer.

As mentioned, the neural network of our research includes input, hidden, output layers, weight, bias, and activation functions. Weight and bias are randomly assigned. The input values are multiplied by the weights and then the bias value is added to their sum. Now, the output is created by using active function. Because the values of the weights are given randomly, they must be changed between runs so that the final output is close to the real value. In fact, learning is done. In the first layer, we have 59 inputs which are features extracted by GLCM. In the hidden layer, we have two layers where there are 10 neurons in each layer, and it performs the processes related to the hidden layer. In the last layer, we have an output that contains the probability matrix of the input belonging to each of the classes. The sigmoid function is used to calculate the output. We have used backpropagation to train the neural network. Also, in the result section, we will say that the cross-validation method was used to validate the diagnosis.

**Figure 4:**
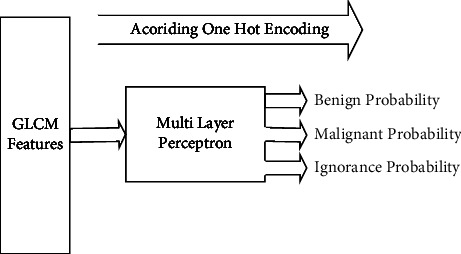
Input and output of our MLP neural network.

According to the above description, the data from all three classes are given as input to the neural network. The output corresponding to each class is considered according to the one hot encoding Figure 4. By GLCM feature extraction from the input image, based on training, the output is determined. We now have a matrix of the probability of belonging to benign, malignant, and ignorant classes per image.

**Figure 5:**
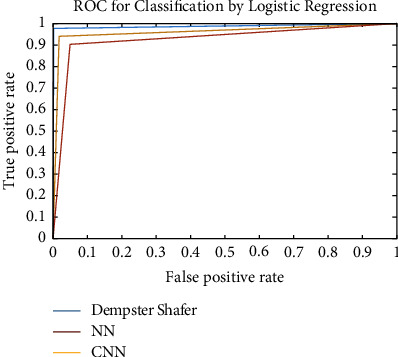
ROC diagram.

**Figure 6:**
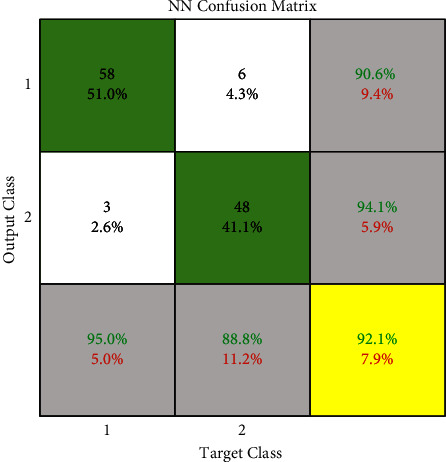
MLP confusion matrix.

By obtaining the output from this step, the accuracy of neural network detection, by maximizing the probability of all three classes, we have achieved an accuracy of 92.2% in class 1, or benign and 94.1 in class 2, or malignant. The ROC and the confusion matrix of this method are shown in Figures5 and 6.

### 3.8. Convolutional Neural Network

**Figure 7:**
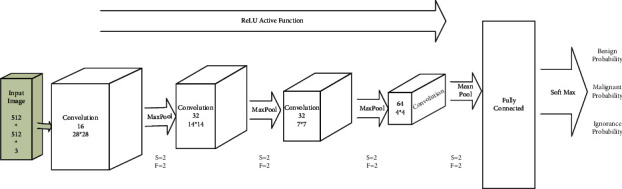
The proposed CNN neural network structure.

Undoubtedly, recent success in deep learning is due to the use of CNN. This neural network consists of one or more layers of convolution that are entirely connected to the upper layer. This method also uses closed weights and merged layers. Compared to other deep neural network architectures, this architecture showed better results in image and speech applications. They are also easier to train than other standard deep-feed neural networks. A few parameters for estimation make them a helpful architecture. In general, a convolutional neural network consists of three main layers: the convolutional layer, the pooling layer, and the fully connected layer, which have different duties for different layers. There are two stages in each convolution neural network: feedforward and backpropagation for training [52]. In the beginning, the input image enters the deep neural network and then multiplies the points between the input and the parameters of each neuron and convolution operation in each layer. After calculating the network output, in order, the parameters related to network training are used to calculate its error rate. In the next step, based on the calculated error value, the backpropagation stage begins. The gradient of each parameter is calculated according to the chain rule, and all neural network parameters change, according to the effect they have on the error created in the network. After updating the parameters, the forward-feed phase begins, and after a specific number of iterations, the training ends. The structure of our proposed convolutional network is shown in Figure 7 and Table 1. As can be seen, 20 layers are used as follows.

**Figure 8:**
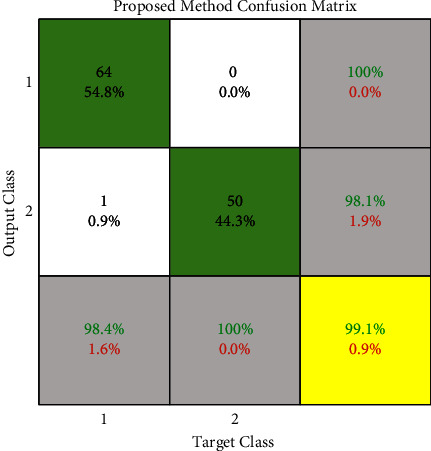
Dempster–Shafer confusion matrix.

Figures 5 and 8 show the ROC and confusion matrix of our proposed convolutional neural network. Moreover, as can be seen, we achieved 98% accuracy in class 1 or malignant and 95.3 accuracies in class 2 or malignant.

### 3.9. Dempster–Shafer Theory

Uncertainty is a challenge that always exists as a negative factor in decisions. Therefore, some system parameters cannot be specified correctly [53]. Over the years, various mathematical models have been proposed to study system uncertainty, and attempts have been made to reduce uncertainty. There are two types of uncertainty: epistemic and aleatory [54]. Aleatory uncertainty is related to the variety of events in nature and refers to the randomness of its observations. It is known as external uncertainty, intrinsic uncertainty, and random uncertainty. Epistemic uncertainty or knowledge uncertainty is the state of knowledge about a physical system and modeling uncertainty. This uncertainty is identified by functional uncertainty, internal uncertainty, and mental uncertainty [55]. There are several ways to display epistemic uncertainty, but since Dempster–Shafer theory can well control uncertainty, in the field of evidence reasoning [56–58], complex evidence theory [59, 60] has been extended. Let us now explain Dempster–Shafer theory. Demonstrator Shafer is one of the data synthesis methods proposed by Dempster in 1967 [61]. In 1976, the development of the Dempster algorithm was done by Shafer [62]. Classical probability theories cannot show ignorance. Using Dempster–Shafer, mass functions can be combined in different ways for probabilities in data mining. In the following, we will introduce this theory and methods of combining information from several different sources. The hypothesis space is considered as Θ {*H*_1_, *H*_2_,…, *H*_*n*_} which the condition of relation (6) applies:

(6) $$\begin{array}{c} \left(H_{i}\cap H_{j}=\emptyset ,\forall i\neq j\right)\text{.} \end{array}$$

The focal space of the hypothesis space is considered a relation:

(7) $$\begin{array}{c} 2^{\Theta }=\left\{\emptyset ,H_{1},H_{2},\ldots ,H_{n},H_{1}\cup H_{2},\ldots ,H_{i}\cup H_{j},H_{k}\cup H_{l}\cup \ldots \cup H_{n},\ldots \Theta \right\}\text{.} \end{array}$$

Two or more mass functions can be combined. The combination of hypotheses is shown in relations (8)–(12):

(8) $$\begin{array}{c} m=m_{1}\;⊕\;m_{2}\ldots ⊕\;m_{n}\text{,} \end{array}$$

(9) $$\begin{array}{c} \left(H_{i}\cap H_{j}=\emptyset ,\forall i\neq j\right)\text{,} \end{array}$$

(10) $$\begin{array}{c} m\left(A\right)=K.\underset{A_{i}\cap B_{j}=A}{\sum }m_{s_{i}}\left(A_{i}\right).m_{s_{i}}\left(B_{j}\right)\text{,} \end{array}$$

(11) $$\begin{array}{c} K=\frac{1}{1-k}\text{,} \end{array}$$

(12) $$\begin{array}{c} k=\underset{A_{i}\cap B_{j}=A}{\sum }m_{s_{i}}\left(A_{i}\right).m_{s_{i}}\left(B_{j}\right)\text{.} \end{array}$$

As mentioned above, our assumptions in this method fall into three classes: benign, malignant, and ignorant. Ignorance means that when the system examines the input image, the features of the cancerous mass are very close to both the benign tumor class and the malignant tumor class. So make the decision very difficult.  Table 2 is created by equation (7). It shows the different positions of the above three classes together to calculate *m* and *k*. *m* and *k* are obtained according to the relation (8)–(12). Then we combine the information obtained from two different sources, MLP and CNN, using equations (7) to (12) by the Dempster–Shafer algorithm. After combining the information obtained from two different sources by Dempster–Shafer theory, Table 3 shows the results. Figures 5 and 8 show the ROC diagram and the confusion matrix of the proposed method.

## 4. Results and Discussion

**Figure 9:**
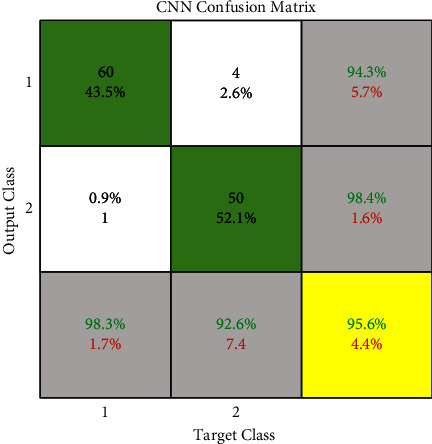
CNN confusion matrix.

We use the cross-validation method to evaluate. In this way, we have divided the data into five categories. Each time, four groups were randomly used for training and one group for testing. The evaluation was performed on 64 samples from the benign class and 51 samples from the malignant class from the MIAS dataset. The test data related to the benign class and the probability of belonging are considered in the first category. In the second category, the test data related to the malignant class and its probability are considered. We now discuss about the ROC, confusion matrix, and the comparison diagrams of the two classes.  Figure 5 shows the ROC of the MLP with texture features, CNN, and the proposed method for the benign and malignant classes. Figures 6–9 show the confusion matrix of MLP with texture features, CNN, and the proposed method for the benign and malignant classes.

**Figure 10:**
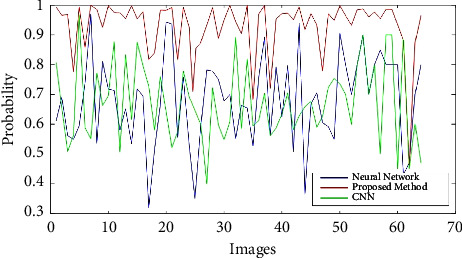
Diagram of all three methods in one frame for class 1 or benign.

**Figure 11:**
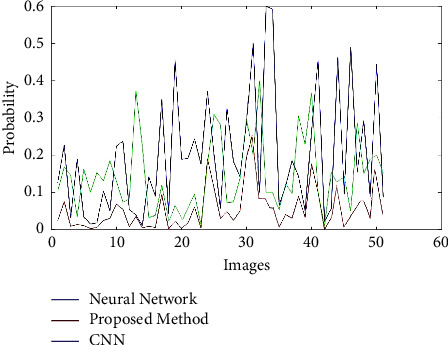
Diagram of all three methods in one frame for class 2 or malignant.

Finally, we draw diagrams of all three methods in one frame, for both benign and malignant classes, in Figures 10 and 11. Also, Table 3 shows the accuracy, sensitivity, and specificity separately by method and class.

As shown in Figure 10, the yellow diagram is related to the deep neural network method, and the blue diagram is related to the neural network class with GLCM features. The blue diagram is related to the proposed Method. The horizontal axis of the diagram shows the samples. Wherever the graph is closer to one, the probability of a correct diagnosis is higher. In Figure 11, which is related to class 2 or malignant, unlike Figure 8, wherever the graph is closer to zero, it means that the probability of a correct diagnosis is higher.

The main comparison criterion for the diagnosis and classification of breast cancer is the percent accuracy.  Table 4 shows the results of the comparison of the proposed approach with other previous methods (see Table 5).

## 5. Conclusion

Accuracy in such processes is far more important than speed. Basically, in the processes related to breast cancer or any cancer, an accurate diagnosis of the type of tumor can play an effective role in treating the disease and its speed of recovery. Uncertainty is a barrier to making the right decision and reduces the accuracy of tumor diagnosis. To solve this problem, we were able to reduce the unknown value in decisions with mathematical relations, increasing the accuracy of the diagnosis. Using two robust classifiers, the tumor output class is the probability of all three classes. By placing these six numbers in Shafer's theory, we obtain three outputs of this method. By finding the maximum, the final class is determined. The accuracy of our method was higher than the previous methods, and we were able to achieve 99.1%. The presence of a mass in the breast area can lead to breast cancer. Early detection and diagnosis of these masses can help in the treatment and maintenance of health. Therefore, intelligent medical diagnostic systems should be developed as a standalone system or as a physician's assistant for providing opinions. Many types of research have been done in recent years for breast cancer diagnosis based on mammography, MRI, and ultrasound images. The disadvantage of most existing methods is the incorrect classification of the masses due to uncertainty in the problem. The proposed approach of this research is to overcome uncertainty and try to reduce ignorance of the problem by using mathematical relations. Using Dempster–Shafer theory, the results based on image processing and machine learning were obtained from two different sources: multi layer perceptron, and deep neural network. After combining the results, we achieved higher accuracy than the previous methods. The obtained classification results in terms of accuracy as evaluation criteria represented that the proposed method has 99.10% accuracy, 100% specificity, and 98.4 sensitivity, which gained a better performance than current methods.

In this research, although good results were obtained, there are also limitations that we express. We need proper and valid evidence to start working, and the evidence used must be completely independent of each other. There are no strict guidelines for the exact design of such systems. Also, the need for tools and calculations determine the amount of belonging to each class and ignorance.

One of the main findings of the research can be mentioned as the negative effect of ignorance on the increase in the error rate. The more ignorance in the problem, the lower the accuracy. Also, the independence of different sources (different methods of classification) is also very important in order to make different diagnosis. By calculating the percentage of the sample belonging to each class and also calculating the ignorance, according to Demester–Shaffer theory, we can reduce the ignorance value and achieve a higher accuracy. This idea can be used in all diagnostic and classification problems.

## Figures and Tables

**Table 1:** Review some of the above methods.

Reference	Method	Advantages	Disadvantages
[16]	Using convolutional neural network and its combination by the multiscale method	Accurate identification of the tumor area	Insufficient accuracy in tumor diagnosis and long run time
[32]	Designed a system for the initial diagnosis, examination, and treatment of breast cancer, combining the features via CNN and the random forest	Improved accuracy rate, precision, and sensitivity	Accuracy still needs to be improved
[35]	Proposed a deep convnet + SVM hybrid network with an accuracy of 97.8%	Proper image noise reduction and proper accuracy	Accuracy can be improved and high computational complexity
[33]	Tested 14 different neural networks on several db and concluded that CNN was slightly more accurate than MLP. CNN alexnet	Tested several various neural networks and db to find the best strap class classifier	Lack of review methods of noise reduction and segmentation
[36]	Comparing random forest, SVM, decision tree, and logistic regression methods and finding the best machine learning algorithms	Study of machine learning methods in diagnosing breast cancer and demonstrate SVM better than other methods	Lack of review methods of noise reduction and segmentation-accuracy can be improved
[37]	Three different structures of CNN and 275,000 images are used to detect breast cancer automatically. The proposed architecture, which was more profound and consisted of five layers	Proposed a more profound architecture for CNN to increase accuracy from	Accuracy is still low and needs to be improved
[17]	Regional growth segmentation method along with the cellular neural network comes with a specified threshold improved classification parameters based on the cellular neural network on the genetic algorithm in mammography images	Improved accuracy rate, precision, and sensitivity compared to other methods such as Naive Bayes, random forest algorithm, support vector machine, and K-nearest neighbor	High computational complexity and extended run time in tumor diagnosis
[18]	Noise removal in the image with background parenchymal enhancement techniques in MRI images	Improved noise reduction in MRI images	Failure to detect the tumor area
[19]	Review article	Study of intelligent methods in diagnosing breast cancer and demonstrate neural network function better than other methods	Lack of review methods of noise reduction and segmentation before feature extraction and classification
[20]	Diagnosis of breast tumors with the use of mammography microarray images	Improve tumor diagnosis accuracy	Incorrect detection of the area-tumors and masses existence of noise in images after preprocessing
[39]	Classify mammographic images to diagnose breast cancer by backpropagation neural network	Diagnosis of the tumor area	Insufficient accuracy in the type of tumor, low processing speed, high computational complexity
[21]	Review article	Study of intelligent methods in diagnosing breast cancer	Lack of review methods of noise reduction and segmentation before feature extraction and classification
[22]	Classification and diagnosis of tumors by Naive Bayes method	Improved accuracy rate, precision, and sensitivity	High computational complexity and extended run time
[23]	Adaptive intelligent decision system for breasts cancer diagnosis from mammography images with a regression approach	Ability to detect tumors and estimate the amount of life of the person after the state tumorous and nonhumorous and exact diagnosis area of the tumor	Insufficient accuracy in tumor diagnosis and high computational complexity
[24]	Diagnosis of breast cancer from mammography images based on features analysis and preprocessing by using an optimal classifier	Improved accuracy rate, precision, and sensitivity diagnosis of benign and malignant tumors	High computational complexity and extended run time
[25]	Diagnosis of breast cancer from mammographic images using preprocess and then use smoothing and thresholding to extract features then using window operations finding min-max with minor variance for diagnosis tumorous and tumor size in the image based on morphological operations and an image gradient technique	Improved accuracy rate, precision, and sensitivity exact diagnosis area of the tumor	High computational complexity and extended run time
[26]	Review article	Study of intelligent methods in diagnosing breast cancer	Lack of review methods of noise reduction and segmentation before feature extraction and classification
[27]	Evaluation of compton camera performance with SI/CZT lens to detect breast tumors using Monte Carlo method	Accurate identification of the tumor area in two- and three-dimensional images	Lack of separation and classification of benign and malignant tumors and failure to evaluate the results
[28]	Use three different deep learning architectures, including Google net, veggie, and Resnet, to diagnose and classify breast cancer from mammography images	Improve the diagnosis and classification of benign and malignant tumors and determine the exact area of the tumor	Not specifying the structure of deep learning in hidden layers including fully connected layers, type of pooling layers, and convolution layer and has long computational complexity and high execution time in diagnosis and classification
[29]	Detection and classification of breast tumors from mammographic images by segmentation based on k-means and surf algorithms and combined classification support vector machine with deep learning	Improve and determine the exact area of the tumor	Lack of a classification of benign and malignant tumors and lack of comparison of the proposed approach with the old methods presented in the scope of deep learning
[30]	Use of the finite element approach	Improve the diagnosis and classification of benign and malignant tumors and determine the exact area of the tumor	Lack of comparison of the proposed system with the old methods presented in the scope of deep learning
[31]	Use of optimal group training	Accurate identification of the tumor area in two- and three-dimensional images	Lack of separation and classification of benign and malignant tumors and failure to evaluate the results

**Table 2:** The proposed CNN neural network structure.

Layer nos.	Layer names	Description
1	Image input	512 ∗ 512 ∗ 3
2	Convolution	16 28 ∗ 28 convolutions with stride [11] and padding “same”
3	Batch normalization	Batch normalization
4	ReLU	ReLU
5	Max pooling	2 ∗ 2 Max pooling with stride [21] and padding [ 0 0 0 0]
6	Convolution	32 14 ∗ 14 convolution with stride [11] and padding “same”
7	Batch normalization	Batch normalization
8	ReLU	ReLU
9	Max pooling	2 ∗ 2 Max pooling with stride [21] and padding [ 0 0 0 0]
10	Convolution	32 7 ∗ 7 convolution with stride [11] and padding “same” and padding “same”
11	Batch normalization	Batch normalization
12	ReLU	ReLU
13	Max pooling	2 ∗ 2 max pooling with stride [21] and padding [ 0 0 0 0]
14	Convolution	64 4 ∗ 4 convolution with stride [11] and padding “same”
15	Batch normalization	Batch normalization
16	ReLU	ReLU
17	Max pooling	2 ∗ 2 mean pooling with stride [21] and padding [ 0 0 0 0]
18	Fully connected	100 fully connected layers
19	Soft Max	Soft Max
20	Classification out put	Cross entropyex

**Table 3:** Combination of MLP and CNN classification information.

Neural network and GLCM Deep neural network			
	Benign class prediction using neural network and GLCM method	Malignant class prediction using neural network and GLCM method	Ignorance class prediction using neural network and GLCM method
Predicting benign class with neural network and deep neural network	Benign probability	Contradiction probability	Contradiction probability
Predicting malignant class with neural network and deep neural network	Contradiction probability	Malignant probability	Contradiction probability
Predicting ignorance class with neural network and deep neural network	Contradiction probability	Contradiction probability	Ignorance probability

**Table 4:** Accuracy, sensitivity, and specificity results obtained in single and combined modes.

Class-method	The accuracy obtained from MLP neural network with GLCM features (%)	The accuracy obtained from deep neural network CNN (%)	Accuracy obtained from combining the results using Dempster–Shafer theory (%)	Final accuracy in benign and malignant images (%)	Final sensitivity in benign and malignant images (%)	Final specificity in benign and malignant images (%)
Class 1-benign	92.2	95.3	98.4	99.1	98.4	100
Class 2-malignant	94.1	96	100			

**Table 5:** Comparison of the proposed approach with recent methods in terms of accuracy.

Reference	Accuracy (%)
Ekici and Jawzal [14]	98.95
Toğaçar et al. [15]	98.59
Yektaei et al. [16]	97.3
Khalilabad et al. [20]	95.45
Kaymak et al. [39]	70.40
Mohebian et al. [31]	85
Karabatak [22]	98.54
Wang et al. [23]	97.10
Rouhi et al. [17]	96.47
Chabert et al. [34]	77
Mahmood et al. [35]	97.8
Naji et al. [36]	97.2
Alanazi et al. [37]	87
Naseem et al. [40]	98.83
Kavitha et al. [41]	98.5
Sadia et al. [43]	97.7
Mangukiya et al. [44]	98.24
Proposed method	99.10

## Data Availability

The data used in this study are available and can be provided over the emails querying directly to the corresponding author (ali.nodehi@gorganiau.ac.ir).
